# The Association Between Obesity and the Development and Severity of Obstructive Sleep Apnea: A Systematic Review

**DOI:** 10.7759/cureus.69962

**Published:** 2024-09-22

**Authors:** Mohamad Ahmad Alenezi, Shahd Alabdulathim, Sultan Abdullah Mutyi Alhejaili, Zahra Abdulelah A Al Sheif, Kade Khalid Aldossari, Jana Ibrahim Bakhsh, Faisl Mohammed Alharbi, Abdulaziz Abdullah Yousef Ahmad, Razan Muidh Aloufi, Hanan Mushaeb

**Affiliations:** 1 Public Health, Maternity and Children Hospital, Ministry of Health, Arar, SAU; 2 Dermatology, King Faisal University, Al Hofuf, SAU; 3 Medicine and Surgery, Tibah University, Madinah, SAU; 4 Obstetrics and Gynecology, Qatif Central Hospital, Qatif, SAU; 5 Medicine and Surgery, King Saud University, Riyadh, SAU; 6 Medicine and Surgery, King Abdulaziz University Hospital, Jeddah, SAU; 7 Medicine and Surgery, Qassim University, Buraidah, SAU; 8 Internal Medicine, King Salman Bin Abdulaziz Medical City, Madinah, SAU; 9 Medicine and Surgery, Almaarefa University, Riyadh, SAU; 10 Medicine, King Abdulaziz University, Jeddah, SAU

**Keywords:** apnea-hypopnea index, body mass index, obesity, obstructive sleep apnea, osa, sleep apnea severity, systematic review

## Abstract

Obesity has reached epidemic proportions globally, accompanied by a parallel rise in the incidence of obstructive sleep apnea (OSA). The systematic study aims to assess the association between obesity and the onset and severity of OSA. A comprehensive computerized search of pertinent databases was done to find studies that fit the inclusion requirements. A comprehensive search was carried out on PubMed, SCOPUS, Science Direct, Systematic Library, and Web of Science to locate relevant material. Our data included 12 trials with 4095 participants, and 1456 (35.6%) were men. In individuals who were obese, the prevalence of OSA varied from 12.6% to 88.9%, with a total prevalence of 1291 (31.5%). One major factor that determined the level of OSA was obesity. It was consistently discovered by studies that there was a positive correlation between body mass index (BMI), and measures such as the Apnea-Hypopnea Index (AHI) are crucial in determining the extent of OSA. Besides, it was also observed that these comorbid conditions made OSA more severe and difficult to manage. Variability in findings suggests the influence of additional factors such as age, sex, and ethnicity on the obesity-OSA relationship. This comprehensive study offers strong evidence that OSA development and severity are significantly influenced by fat. The results emphasize the significance of weight control, especially for obese people, in treating and preventing OSA.

## Introduction and background

Obstructive sleep apnea (OSA) is a sleep disorder characterized by repeated interruptions in breathing during sleep due to the relaxation of throat muscles. These interruptions can lead to decreased oxygen levels in the blood and cause frequent awakenings, resulting in fragmented sleep [1]. Obesity is a medical condition characterized by an excessive accumulation of body fat that may impair health. It is typically assessed using the body mass index (BMI), which is calculated by dividing a person's weight in kilograms by their height in meters squared. A BMI of 30 or higher is generally classified as obesity [2].

Obesity increases the likelihood of developing OSA primarily through anatomical changes. Obesity contributes to OSA primarily by increasing the amount of fatty tissue in the neck and throat area, which can obstruct the airway during sleep [2]. Additionally, excess weight can lead to increased pressure on the diaphragm, making it harder to breathe. This combination of factors increases the likelihood of airway collapse, resulting in interrupted breathing episodes during sleep [3].

Moreover, there exists a robust association between the extent of obesity and the intensity of OSA. According to a study in 1993 published in *New England Journal of Medicine*, the risk of developing severe OSA may increase two to four times for every unit increase in BMI [4]. Higher levels of obesity can worsen airway blockage and cause other physiological changes, including inflammation and elevated intra-abdominal pressure, which can affect lung function and cause sleep problems [5].

Additionally, obesity can affect the effectiveness of conventional treatments for OSA. A therapy known as CPAP, or continual positive airway pressure, is widely used to treat moderate to severe instances of OSA but may be less effective in obese patients if the underlying anatomical issues are not addressed. Weight loss interventions often lead to significant improvements in OSA symptoms, highlighting the importance of managing obesity as a dual approach to mitigate the effects of this disorder [6].

Obesity has reached epidemic proportions globally, with significant health implications. OSA is a prevalent and often underdiagnosed disorder associated with numerous comorbidities. Since obesity and OSA have a significant correlation, it is imperative to comprehend the specifics of this link to develop preventive, diagnostic, and management techniques that work. Despite the growing body of research on obesity and OSA, there is a need for a thorough and methodical analysis to evaluate the overall strength of the available data. The study aims to identify and analyze relevant research on the connection between obesity and OSA.

## Review

Methods

The study was conducted by following the guidelines of the Preferred Reports for Systematic Reviews and Meta-Analyses (PRISMA) [7]. Our systematic review conducted a comprehensive analysis of the influence of comorbidities on trauma patient outcomes. To find relevant English-language studies that investigate the impact of pre-existing medical conditions on the impact of obesity on the severity and development of OSA, PubMed, Web of Science, SCOPUS, and Google Scholar were searched extensively using keywords "Obesity, obstructive sleep apnea, OSA, systematic review, meta-analysis, body mass index, BMI, sleep apnea severity, and apnea-hypopnea index." The search technique included terms related to the impact of fat on the development and severity of OSA. After carrying out separate searches, two reviewers located pertinent studies, collected information, and used the proper grading instruments to provide a quality rating for the included research.

Eligibility criteria

Inclusion criteria for the studies involve randomized controlled trials, case-control, cohort, and longitudinal designs, focusing on adult participants (18 years or older) diagnosed with obesity (BMI of 30 kg/m^2^). The research must examine exposure to obesity or weight-related factors and report outcomes related to OSA severity, including measures like the Epworth Sleepiness Scale or the Apnea-Hypopnea Index (AHI), published between 2023 and 2024. A comparison group of non-obese individuals or those with varying obesity levels is required, and studies must be in English.

Exclusion criteria include case series, evaluations, editorials, meta-analyses, and case studies. Participants with other sleep disorders that could confound the relationship between obesity and OSA, as well as studies focusing solely on weight loss without assessing OSA impact, are excluded. Additionally, studies lacking clear OSA definitions, those with inadequate methodological rigor, and research published in languages other than English were considered.

Data extraction

The search results were verified for correctness using Rayyan (QCRI) [8]. The bearing with which the search's titles and abstracts turned up was assessed using the inclusion and exclusion criteria. The study team gave careful consideration to the papers that met the inclusion conditions. Disagreements were resolved by consensus. Key study information was obtained using a pre-established data extraction form. This information included the study location, gender distribution, authors, titles, publication year, participant demographics, and how weight affects the degree and progression of OSA. An objective evaluation tool was developed to examine the potential for bias.

Using data from relevant studies to give a qualitative assessment, summaries of the research findings and elements were created. A qualitative assessment was produced by summarizing the components and study findings.

Risk of bias assessment

The critical assessment standards developed by the Joanna Briggs Institute (JBI) will be used to evaluate the study's quality [9] for research that offers statistics on prevalence. There are nine questions in this tool. A score of one is given for a good reaction, whereas a negative, unclear, or insignificant reaction is given a score of zero. Low, moderate, and high-quality ratings were assigned to the following scores, in that order: below four, between five and seven, and above eight. Disagreements were settled through discussion after researchers evaluated each study individually.

Results

**Figure 1:**
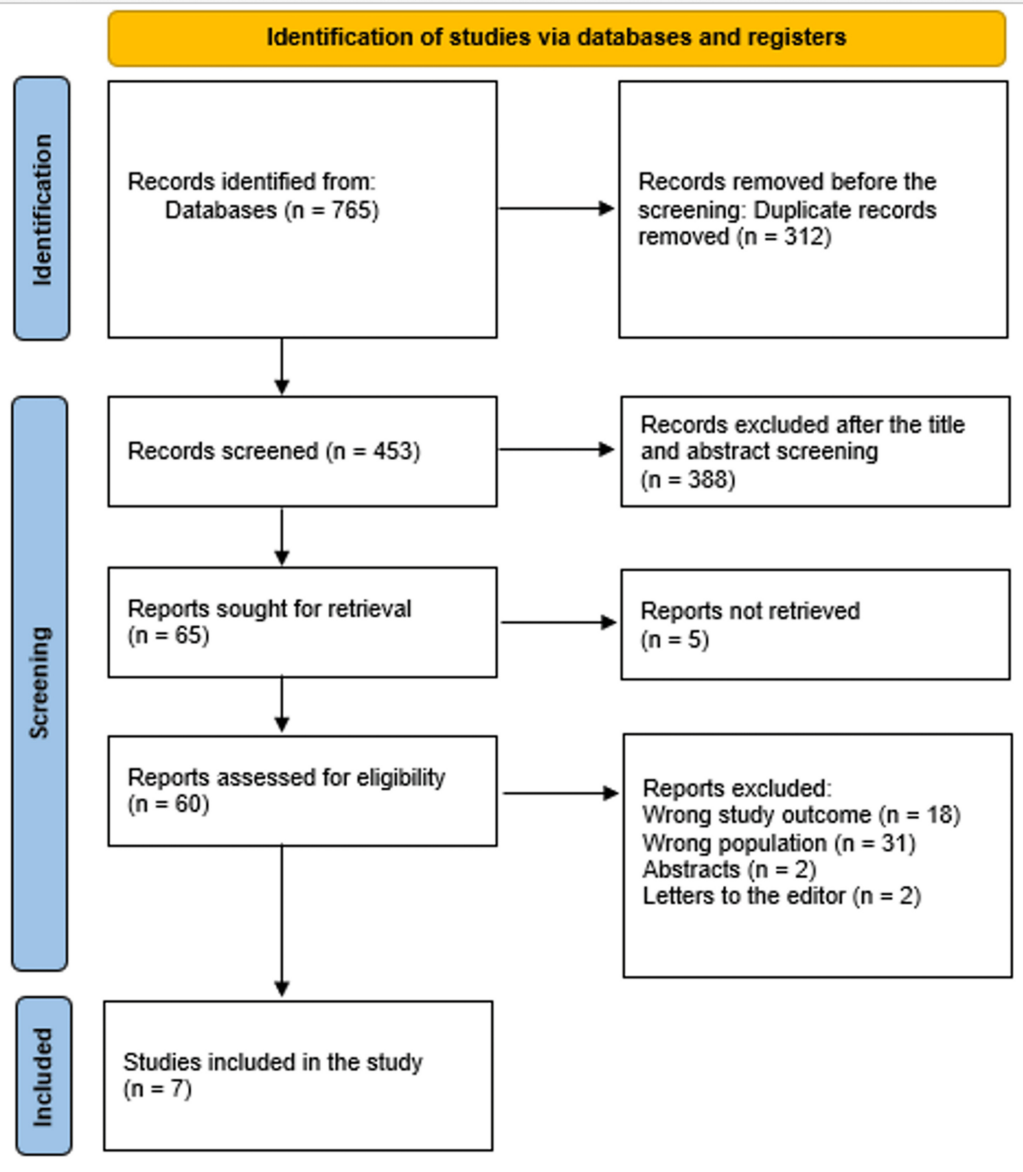
Illustration of the study selection utilizing a PRISMA diagram PRISMA: Preferred Reporting Items for Systematic Reviews and Meta-Analyses

The method used to choose the literature is shown in a diagram in Figure 1.

The socio-demographic data collected from the studies published are displayed in Table 1. Twelve studies with 4095 people were included in our data [10-16], and 1456 (35.6%) were males. Four studies were retrospective cohorts [11,14-16], two were cross-sectional [10,12], and one was a retrospective observational study [13]. These studies were conducted in various countries, including Saudi Arabia, the Netherlands, India, Israel, China, and Switzerland. The number of participants ranged from 143 to 2,441, with a mean age ranging from 9.2 to 53.7 years old.

**Table 1:** Sociodemographic parameters of the involved populations NM: not mentioned

Study	Study design	Country	Participants	Mean age	Males (%)
AlAteeq et al., 2024 [10]	Cross-sectional	Saudi Arabia	378	NM	79 (20.9%)
van Ede et al., 2024 [11]	Retrospective cohort	The Netherlands	2,441	42.1 ± 11.9	595 (24.4%)
Dixit et al., 2024 [12]	Cross-sectional	India	33	49.2 ± 5.8	9 (24.2%)
Brikman et al., 2024 [13]	Retrospective observational study	Israel	175	40.4 ± 5.6	60 (34.3%)
Hao et al., 2023 [14]	Retrospective cohort	China	207	NM	72 (34.8%)
Hao et al., 2023 [15]	Prospective cohort	China	718	53.7 ± 10.8	612 (85.2%)
Agossou et al., 2023 [16]	Prospective cohort	Switzerland	143	NM	29 (20.3%)

Clinical outcomes

The clinical information is displayed in Table 2. Studies employed various diagnostic tools, including the SLEEP-50 questionnaire, the OBES questionnaire, the Epworth Sleepiness Scale (ESS), and polysomnography (PSG). The obesity cut-off point varied across studies, with most using a BMI of >30 kg/m^2^. The prevalence of OSA in obese patients ranged from 12.6% [13] to 88.9% [14], with a total prevalence of 1291 (31.5%) and higher rates generally observed in studies using PSG as the diagnostic tool [12,14,16]. There were different populations that had varied levels of obesity, OSA severities, and other obesity-related comorbidities. One major factor that determined the level of OSA was obesity. It was consistently discovered by studies that there was a positive correlation between BMI, and measures such as the AHI are crucial in determining the extent of OSA. Besides, it was also observed that these comorbid conditions made OSA more severe and difficult to manage. Variability in findings suggests the influence of additional factors such as age, sex, and ethnicity on the obesity-OSA relationship.

**Table 2:** Clinical parameters and outcomes of the comprised research OSA: obstructive sleep apnea; OSAS: obstructive sleep apnea syndrome; PSG: polysomnography; ESS: Epworth sleepiness scale; OBES: obesity; BMI: body mass index; ACS: acute coronary syndrome; NM: not mentioned; JBI: Joanna Briggs Institute

Study ID	OSA diagnostic tool	Obesity cut-off point	Mean BMI (kg/m^2^)	Prevalence of OSA (%)	Main outcomes	JBI
AlAteeq et al., 2024 [10]	SLEEP-50 questionnaire	NM	NM	67 (17.7%)	Obese adults have a significant prevalence of sleep problems. Multiple co-occurring sleep disorders were observed in a considerable number of persons with sleep disorders, and there was a significant correlation between these conditions and obesity and overweight.	Moderate
van Ede et al., 2024 [11]	The OBES-questionnaire	>30	42.4 ± 4.5	422 (17.3%)	Because years of OSAS without a diagnosis were not taken into account, a negative correlation was discovered for OSAS incidence. Furthermore, there was no correlation found between OBES and blood markers associated with poor metabolism when obesity-related comorbidities were absent.	Moderate
Dixit et al., 2024 [12]	PSG	NM	32 ± 2	5 (15.2%)	It was discovered that the proportion of obese patients with obstructive sleep apnea-hypopnea syndrome was comparable to other studies carried out in comparable environments.	High
Brikman et al., 2024 [13]	NM	>30	42.1 ± 6.3	22 (12.6%)	NM	High
Hao et al., 2023 [14]	PSG	>30	48.4 ± 12.6	184 (88.9%)	Males were the only significant contributory factor and females were the protective factor against severe OSA in individuals with a BMI > 35 kg/m^2^.	Moderate
Hao et al., 2023 [15]	ESS	>25	31.0 ± 2.5	485 (76.5%)	Given the frequent coexistence of obesity and OSA, it is crucial to take into account their proportional risk of cardiovascular events as well as how they interact. According to this study, OSA considerably raised the chance of cardiovascular events following ACS, even in patients who were not obese.	Moderate
Agossou et al., 2023 [16]	PSG	>30	45.2 ± 8.7	106 (74.1%)	NM	Moderate

While most studies found a positive association between obesity and OSA, the strength and nature of this relationship varied. For example, one study found a significant correlation between obesity and sleep disorders, while another found no correlation between obesity and blood markers associated with poor metabolism in the absence of obesity-related comorbidities.

Discussion

This study found that among overweight and obese patients (BMI > 25), the total prevalence of OSA was 1291 (31.5%), with a range of 12.6% [13] to 88.9% [14]. In the US, adult males with OSA have a prevalence of 3% to 7%, whereas adult females have a prevalence of 2% to 5% [17]. Between 0.3% and 0.4% of OHS patients are thought to have existed in both Europe and the US [18,19].

We found that there were different populations that had varied levels of obesity, OSA severities, and other diseases. One major factor that determined the level of OSA was obesity. It was consistently discovered by studies that the relationship between BMI and AHI was favorable which is an important measure for diagnosing the severity of OSA. Besides, it was also observed that these comorbid conditions made OSA more severe and difficult to manage. Variability in findings suggests the influence of additional factors such as age, sex, and ethnicity on the obesity-OSA relationship. Liu et al. reported that patients with OSA alone experienced hypoxia and nocturnal apnea; however, OSA patients with OHS experienced a greater drop in AHI and mean SpO_2_. Patients with OSA and OHS frequently accumulate fat, and those with OSA and OHS typically have larger neck circumferences [20]. Weight loss can dramatically lessen the severity of OSA, whether it comes from medication, bariatric surgery, or lifestyle changes. However, each person responds to these approaches differently, and maintaining weight loss is still difficult. Moreover, the review indicates that comorbidities are associated with obesity [21].

Subsequent studies ought to concentrate on pinpointing the fundamental processes that associate obesity with OSA, namely the role played by adipokines, inflammation, and metabolic variables in the development of the condition. To evaluate the long-term impact of weight control methods on the severity of OSA and the outcomes of patients, longitudinal studies are required. Furthermore, studies should investigate customized strategies for treating OSA that take into account an individual's body fat distribution, genetic risk, and the existence of comorbidities linked to obesity.

To manage individuals with obesity-related OSA, more research is required to determine the effectiveness of combination therapy, such as CPAP, in addition to weight loss programs. Additional research should examine the impact of novel medications on the progression of OSA, such as cutting-edge pharmacotherapies that target obesity. Understanding the behavioral, psychological, and socioeconomic factors that prevent persons with OSA from effectively maintaining their weight will be crucial to developing more effective and long-lasting therapies.

There are various limitations to this systematic review. First, the generalizability of the results might be limited by the differences in research designs, populations, and outcome measures among the included studies. Furthermore, the majority of research relied on observational data, which can introduce bias and make it more difficult to prove a link between OSA and obesity.

## Conclusions

In summary, this detailed examination of the relationship between obesity and OSA not only elucidates the physiological mechanisms through which excess fat contributes to the onset and exacerbation of this condition but also underscores the critical role that weight management plays in mitigating its impacts. By showcasing the clear correlation between BMI and the prevalence of OSA, the findings highlight the importance of targeted interventions focused on weight control as a viable strategy for reducing OSA risks, particularly for individuals at higher risk due to obesity. As we move forward, it is essential to integrate these insights into public health strategies, ensuring that both healthcare providers and patients recognize the transformative potential of weight management in improving sleep-related outcomes and overall health. Furthermore, this research opens up pathways for future investigations into additional lifestyle changes and therapeutic options that could enhance OSA treatment, ultimately fostering a more holistic approach to addressing this pervasive sleep disorder.
